# Confirmed Activity and Tolerability of Weekly Paclitaxel in the Treatment of Advanced Angiosarcoma

**DOI:** 10.1155/2016/6862090

**Published:** 2016-02-25

**Authors:** Gaetano Apice, Antonio Pizzolorusso, Massimo Di Maio, Giovanni Grignani, Vittorio Gebbia, Angela Buonadonna, Annarosaria De Chiara, Flavio Fazioli, Giampaolo De Palma, Danilo Galizia, Carlo Arcara, Nicola Mozzillo, Francesco Perrone

**Affiliations:** ^1^Istituto Nazionale Tumori IRCCS “Fondazione G. Pascale”, Via Mariano Semmola, 80131 Napoli, Italy; ^2^Institute for Cancer Research and Treatment, Strada Provinciale, km 3.95, Candiolo, 10060 Turin, Italy; ^3^Medical Oncology Unit, La Maddalena Hospital, Via San Lorenzo Colli 312/d, 90146 Palermo, Italy; ^4^Departments of Radiation Oncology and Medical Oncology, CRO, National Cancer Institute, Via Franco Gallini 2, 33081 Aviano, Italy

## Abstract

*Background.* In several prospective and retrospective studies, weekly paclitaxel showed promising activity in patients with angiosarcoma.* Patients and Methods*. Our study was originally designed as a prospective, phase II multicenter trial for patients younger than 75, with ECOG performance status 0–2, affected by locally advanced or metastatic angiosarcoma. Patients received paclitaxel 80 mg/m^2^ intravenously, at days 1, 8, and 15 every 4 weeks, until disease progression or unacceptable toxicity. Primary endpoint was objective response.* Results*. Eight patients were enrolled but, due to very slow accrual, the trial was prematurely stopped and further 10 patients were retrospectively included in the analysis. Out of 17 evaluable patients, 6 patients obtained an objective response (5 partial, 1 complete), with an objective response rate of 35% (95% confidence interval 17%–59%). Of note, five responses were obtained in pretreated patients. In the paper, details of overall survival, progression-free survival, and tolerability are reported.* Conclusions*. In this small series of patients with locally advanced or metastatic angiosarcoma, weekly paclitaxel was confirmed to be well tolerated and active even in pretreated patients.

## 1. Introduction

Angiosarcomas are very rare tumors (incidence < 1/100.000/year) of vascular or lymphatic origin characterized by a clinical heterogeneity in terms of presentation and behavior. This subgroup of sarcomas represent about 1-2% of all soft tissue tumors and can occur in any anatomic site of the body but most commonly originate in the skin of head and neck and in breast area [1].

Although etiology is unknown, several risk factors for angiosarcoma have been described: previous exposure to radiation therapy [2, 3], vinyl chloride [4], chronic lymphedema [5], and prolonged immunosuppression [6, 7].

Regardless of morphology, angiosarcoma is considered as a high-grade tumor [8] and constitutes one of the most aggressive subtypes of soft tissue sarcomas with overall median survival of <4 years. In our experience even angiosarcomas histologically classified as low grade can develop distant metastasis.

Wide surgical resection followed as much as possible by adjuvant radiation therapy is the mainstay of therapy in patients with localized disease [9, 10].

As for other soft tissue sarcomas chemotherapy is still not a standard treatment in adjuvant setting, despite the fact that angiosarcomas develop distant metastasis in up to 50% of cases [11, 12].

Doxorubicin-based chemotherapy remains the first-line standard treatment of metastatic or unresectable angiosarcoma providing a progression-free survival of 3.7–5.4 months and response rate between 40% and 65% [11].

Taxanes have been found effective in patients affected by vascular-derived tumors, such as Kaposi sarcoma [13]. Paclitaxel is potent antiangiogenic drug and, at least* in vitro*, exhibits its efficacy on human endothelial cells at low-dose concentration as well as cytotoxic effect at regular concentration [14–16].

In this paper we report a retrospective series of 17 patients with advanced angiosarcoma treated with weekly paclitaxel in 4 centers of Italian Sarcoma Group.

## 2. Patients and Methods

### 2.1. Patients

The study was originally designed as a prospective, phase II multicenter trial with the aim of assessing activity and toxicity of the weekly schedule of paclitaxel. Patients with histological diagnosis of angiosarcoma, with locally advanced or metastatic disease, and not eligible for surgery or recurrent after previous surgery were eligible for the inclusion in the study. Previous chemotherapy was allowed, but it had to be stopped at least 4 weeks before the inclusion in the protocol. Main exclusion criteria were age younger than 75, performance status worse than 2 according to Eastern Cooperative Oncology Group, other malignant diseases in the previous 5 years (with the exception of nonmelanomatous skin cancer or carcinoma* in situ* of the uterine cervix), and brain metastases or inadequate laboratory values (neutrophils < 2000/mm^3^, platelets < 100000/mm^3^, hemoglobin < 10 g/dL, serum creatinine level > 1.5 x upper normal limit (UNL), sAST or sALT > 1.25xUNL in the absence of liver metastases or >2.5xUNL in the presence of liver metastases, and serum bilirubin > 1.25xUNL in the absence of liver metastases or >1.5xUNL in the presence of liver metastases). The study protocol was approved by the ethical committees of all participating institutions, and all patients prospectively enrolled in the trial provided written informed consent.

### 2.2. Study Treatment

Patients received paclitaxel 80 mg/m^2^ intravenously (IV), at days 1, 8, and 15 every 4 weeks until disease progression or unacceptable toxicity. Standard premedication with dexamethasone and H1 (promethazine) and H2 (ranitidine) receptor antagonists was prescribed by protocol, before each administration of paclitaxel. Chemotherapy could be postponed, at Investigator's discretion, for up to 14 days for persistent hematological toxicity (neutrophils < 1500/mm^3^; platelets < 100000/mm^3^; hemoglobin < 8 g/dL) or persistent nonhematological toxicities grade ≥2. A 25% dose reduction (60 mg/m^2^) for paclitaxel was planned in case of previous grade 4 neutropenia lasting more than 3 days or in case of previous platelets < 50000/mm^3^. After disease progression, there was no predetermined salvage treatment planned by study protocol; however further chemotherapy was allowed at Investigators' discretion.

### 2.3. Assessment Procedures

Patients were evaluated at baseline with a complete history and physical examination, routine hematology and biochemistry, chest X-ray, chest CT scan, abdominal ultrasound, and CT scan (or magnetic resonance or ultrasound) for specific sites of disease.

Tumor response was assessed by repeating instrumental exams every two cycles of chemotherapy, by using RECIST criteria version 1.0 [17].

Toxicity was codified according to National Cancer Institute Common Terminology Criteria (version 2.0). During treatment, routine hematology, biochemistry, and physical examination were performed every 3 administrations of paclitaxel, before the next cycle. Hematology was also repeated before each weekly administration of chemotherapy.

### 2.4. Sample Size and Statistical Analysis

Objective response was the primary endpoint of the trial, and the sample size of the study was determined according to Gehan's two-stage design, based on the requirement of stopping the study at an early stage if the response rate was below 20%, and of estimating the response rate with a standard error less than 0.10 [18]. If no objective tumor response was observed among the first 14 evaluable patients, recruitment of patients would stop, whilst additional (1, 6, 9, or 11) patients had to be included if there were responses (1, 2, 3, or more than 3, resp.) in the first 14 patients.

Median follow-up was calculated according to the reverse Kaplan-Meier technique [19]. Overall survival (OS) was calculated from the date of treatment start to the date of death, or the date of last follow-up for alive patients. Progression-free survival (PFS) was defined as the time from the date of treatment start to the date of disease progression, or the date of death for patients that died without progression, or the date of last follow-up for patients alive and without progression at the end of the study. OS and PFS curves were estimated according to the Kaplan-Meier product limit method.

Statistical analyses were performed with S-PLUS software (S-PLUS 6.0 Professional, release 1, Insightful Corporation, Seattle, WA, USA).

## 3. Results

Between October 2002 and March 2006, 8 patients were enrolled in the prospective trial by 5 Italian institutions. The trial was prematurely stopped, due to very slow accrual, although the planned number of 14 patients had not been reached. Further 10 patients, who started treatment with weekly paclitaxel between April 2003 and November 2011, were retrospectively included in the analysis.

One patient enrolled in the prospective trial has been excluded from the analysis due to lack of postregistration data. Baseline characteristics of the 17 evaluable patients, overall and scattered by prospective versus retrospective group, are summarized in Table 1. Median age was 64 years (range 20–80), and most patients had ECOG performance status 0 or 1. The majority of patients had metastatic disease (13, 76%) and had received previous surgery. All the 7 patients enrolled in the prospective trial were pretreated with chemotherapy for advanced disease, whilst 7 out of 10 patients retrospectively analyzed received paclitaxel as first-line of treatment for advanced disease. Individual characteristics of all the treated patients are listed in Table 2.

Median number of paclitaxel administrations in the 17 evaluable patients was 12 (range, 4–30). Two patients received more than 6 cycles of treatment, stopping because of disease progression after 8 and 10 cycles, respectively. Median number of paclitaxel administrations in the 7 patients enrolled in the prospective study was 8 (range, 6–18). Median number of paclitaxel administrations in the 10 patients enrolled in the retrospective study was 13.5 (range, 4–30).

Median dose intensity of paclitaxel in the 17 evaluable patients was 60 mg/m^2^/week (range, 43–80). Median dose intensity of paclitaxel in the 7 patients enrolled in the prospective study was 60 mg/m^2^/week (range, 44–68). Median dose intensity of paclitaxel in the 10 patients enrolled in the retrospective study was 61 mg/m^2^/week (range, 42–80).

Overall, 6 patients obtained an objective response (5 partial responses, 1 complete response). Objective response rate was 35% (95% confidence interval 17%–59%). Of note, five of the objective responses were obtained in patients already pretreated with chemotherapy. Considering only the 7 patients enrolled in the prospective trial, 2 partial responses were observed (objective response rate 29%, 95% confidence interval 8%–64%).

After a median follow-up of 20.8 months, 14 progressions (82%) and 8 deaths (47%) were recorded in the 17 evaluable patients. In the prospective group, after a median follow-up of 20.8 months, 7 progressions (100%) and 4 deaths (57%) were recorded. In the retrospective group, after a median follow-up of 45.9 months, 7 progressions (70%) and 4 deaths (40%) were recorded.

**Figure 1:**
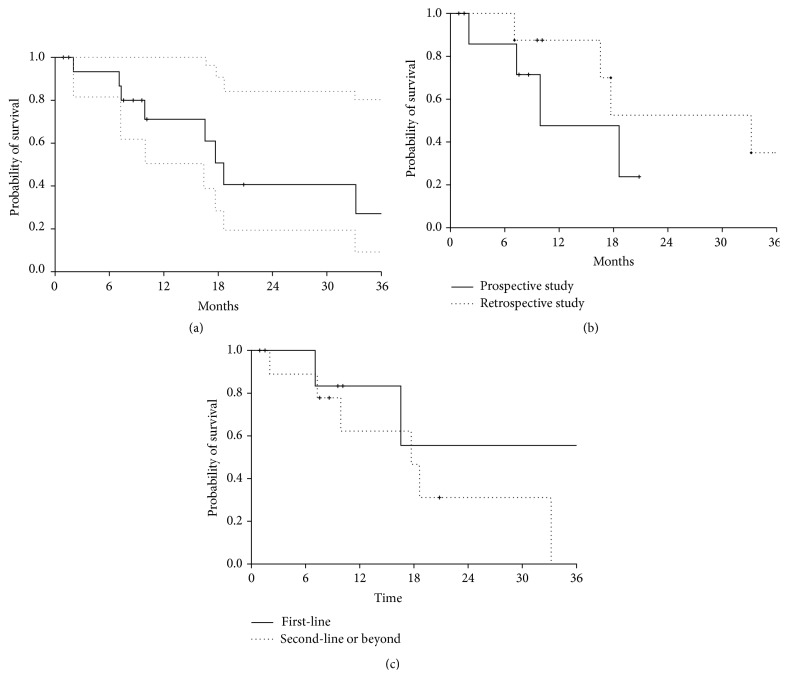
Kaplan-Meier curves of overall survival. (a) Overall survival in the whole series of patients (prospective + retrospective). Dotted lines represent 95% confidence intervals. (b) Overall survival according to type of study: continuous line refers to patients enrolled in the prospective study; dotted line refers to patients included in the retrospective study. (c) Overall survival according to line of treatment: continuous line refers to patients treated with paclitaxel as first-line; dotted line refers to patients receiving paclitaxel as second-line or further line.

Median overall survival was 18.6 months (95% confidence interval (CI) 16.5–n.a.) (Figure 1(a)). In the prospective group, median overall survival was 9.9 months (95% CI 7.3–n.a.), whilst, in the retrospective group, median overall survival was 33.2 months (95% CI 16.5–n.a.) (Figure 1(b)). In patients receiving experimental treatment as first-line, median overall survival was not reached (95% CI 16.5–n.a.), whilst, in patients receiving experimental treatment as second-line or further line, median overall survival was 17.7 months (95% CI 9.9–n.a.) (Figure 1(c)).

**Figure 2:**
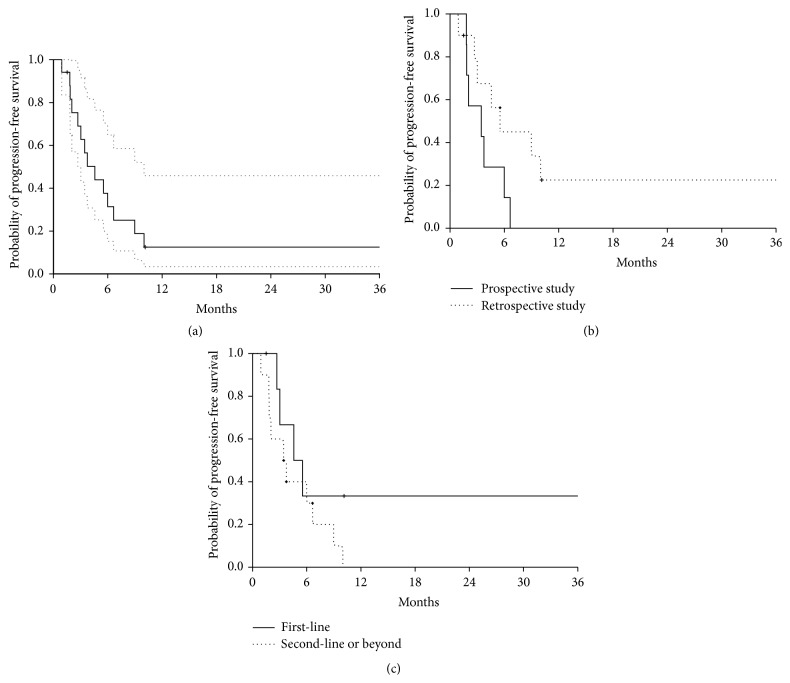
Kaplan-Meier curves of progression-free survival. (a) Progression-free survival in the whole series of patients (prospective + retrospective). Dotted lines represent 95% confidence intervals. (b) Progression-free survival according to type of study: continuous line refers to patients enrolled in the prospective study; dotted line refers to patients included in the retrospective study. (c) Progression-free survival according to line of treatment: continuous line refers to patients treated with paclitaxel as first-line; dotted line refers to patients receiving paclitaxel as second-line or further line.

Median progression-free survival was 4.6 months (95% confidence interval (CI) 2.7–10.0) (Figure 2(a)). In the prospective group, median progression-free survival was 3.5 months (95% CI 1.8–n.a.), whilst, in the retrospective group, median progression-free survival was 5.5 months (95% CI 3.0–n.a.) (Figure 2(b)). In patients receiving experimental treatment as first-line, median progression-free survival was 5.1 months (95% CI 3.0–n.a.), whilst, in patients receiving experimental treatment as second-line or further line, median progression-free survival was 3.6 months (95% CI 1.8–n.a.) (Figure 2(c)).

Mild or moderate anemia was reported during treatment in ten patients (59%), grade 1 in 9 patients and grade 2 in 1 patient. Any grade neutropenia was reported in 4 patients (24%), grade 3-4 neutropenia was reported in 2 patients, and there was no case of febrile neutropenia. Any grade thrombocytopenia was reported in 5 patients (29%), grade 3-4 thrombocytopenia was reported in 2 patients, and there were no relevant bleeding episodes. Mild or moderate asthenia was reported in 6 patients (35%), mild or moderate skin toxicity in 3 patients (18%), grade 1-2 diarrhea in 3 patients (18%), grade 1 constipation in 3 patients (18%), and grade 1-2 nausea or vomiting in 2 patients (12%). Neuropathy was reported in 5 patients (grade 3 in 1, grade 2 in 1, and grade 1 in 3 patients). No severe organ toxicities were described.

## 4. Discussion

In recent years, after the start of our study, several experiences with paclitaxel in patients with advanced or metastatic angiosarcoma have been published [20–23].  Table 3 reports the main characteristics and results obtained in these studies and in our series. The EORTC soft tissue and bone sarcoma group published a retrospective study about the use of paclitaxel in 32 patients [21]. Only 11 patients of this series received a weekly schedule of paclitaxel; the others were treated with the classical, every-3-week schedule. In the whole series, response rate was 62% and median progression-free survival was 7.6 months; however many of the patients were not pretreated with chemotherapy. These results prompted the authors to define paclitaxel as active agent, warranting prospective trials in this setting. Similarly, weekly paclitaxel was associated with promising efficacy in a retrospective analysis of patients treated between 1996 and 2009 in the French Sarcoma Group [23].

In a prospective phase II trial, 30 patients were treated with weekly paclitaxel, at the same schedule tested in our study. In that series of patients (11 pretreated with chemotherapy and 19 not pretreated), weekly paclitaxel produced 18% response rate, a median time-to-progression of 4 months, and a median overall survival of 8 months. Similar to our series, results were considered encouraging also in the subgroup of patients who had already failed previous chemotherapy, with similar progression-free survival compared to those who were treatment naïve.

Recently, a randomized phase II trial testing the addition of bevacizumab to weekly paclitaxel in patients with advanced or metastatic angiosarcoma was presented [24]. In that trial, patients assigned to control arm received paclitaxel 90 mg/m^2^ at days 1, 8, and 15 every 4 weeks, and patients assigned to experimental arm received the same schedule with the addition of bevacizumab. Unfortunately, the addition of the antiangiogenic monoclonal antibody was not associated with any benefit in PFS nor in overall survival.

The retrospective fraction of this study did not allow us to describe exhaustively the adverse events related to chemotherapy. However no important toxicities were reported. There were no cases of febrile neutropenia and no relevant bleeding episodes, whilst grade 3-4 neutropenia was reported in 2 cases and only 1 patient exhibited grade 3 neuropathy. In conclusion, our experience confirms that weekly paclitaxel is well tolerated and active in patients with advanced and metastatic angiosarcomas, even though, in our experience, duration of response was quite short. Further studies of this agent are warranted, also in combination with other drugs. An interesting schedule with taxanes and doxorubicin, an active combination in other tumors, may be a major issue, particularly in neoadjuvant setting, whereas the combination of anthracycline plus ifosfamide can be difficult to administer in many angiosarcoma patients due to the age and clinical conditions.

## Figures and Tables

**Table 1:** Baseline characteristics.

	Prospective group (*n* = 7)	Retrospective group (*n* = 10)	Study population (*n* = 17)
Gender			
Males	3 (43%)	5 (50%)	8 (47%)
Females	4 (57%)	5 (50%)	9 (53%)
Age			
Median (range)	63 (20–74)	68 (46–80)	64 (20–80)
ECOG performance status (3 missing data items)			
0	3 (43%)	2 (29%)	5 (36%)
1	3 (43%)	5 (71%)	8 (57%)
2	1 (14%)	—	1 (7%)
Stage			
Locally advanced	1 (14%)	3 (30%)	4 (24%)
Metastatic	6 (86%)	7 (70%)	13 (76%)
Grading (1 missing data item)			
G1	—	—	—
G2	3 (43%)	4 (44%)	7 (44%)
G3	4 (57%)	5 (56%)	9 (56%)
Previous surgery			
Yes	5 (71%)	9 (90%)	14 (82%)
Previous chemotherapy			
None	—	5 (50%)	5 (29%)
Only adjuvant	—	2 (20%)	2 (12%)
Advanced disease	7 (100%)	3 (30%)	10 (59%)

**Table 2:** Individual characteristics of the 17 patients included in the analysis.

Patient code	Type of study	Gender	Age	PS	Stage	Grading	Previous surgery	Previous chemotherapy	Best response	PFS (months)	OS (months)
1	P	F	35	2	Metastatic	G3	Yes	Yes	PD	0.9	0.9^+^
2	P	M	64	0	Metastatic	G3	No	Yes	PD	10.1^+^	10.1^+^
4	P	M	20	0	Metastatic	G3	Yes	Yes	PR	1.5^+^	1.5^+^
5	P	F	50	1	Metastatic	G2	Yes	Yes	PR	9.0	17.7
6	P	F	63	0	Metastatic	G2	Yes	Yes	PD	10.0	33.2
7	P	F	67	1	Loc. adv.	G3	No	Yes	SD	62.2^+^	62.2^+^
8	P	M	74	1	Metastatic	G2	Yes	Yes	SD	5.5	45.9^+^
1001	R	M	73	1	Loc. adv.	G3	Yes	No	SD	2.7	9.6^+^
1002	R	M	70	1	Metastatic	G3	Yes	Yes	PD	4.6	7.1
1003	R	F	66	0	Metastatic	G2	Yes	Yes	PR	6.6	8.6^+^
1004	R	F	75	0	Metastatic	G3	Yes	Yes	PR	3.0	16.5
1005	R	M	46	1	Metastatic	n.a.	Yes	Yes	SD	3.5	7.6^+^
1006	R	F	61	1	Metastatic	G2	Yes	Yes	CR	1.8	2.0
1007	R	F	62	n.a.	Metastatic	G3	Yes	No	PR	2.0	20.8^+^
1008	R	M	71	n.a.	Loc. adv.	G2	Yes	No	PD	3.8	7.3
1009	R	M	80	1	Loc. adv.	G3	No	No	SD	6.0	9.9
1010	R	F	47	n.a.	Metastatic	G2	Yes	No	SD	1.8	18.6

P: prospective; R: retrospective; M: male; F: female; PS: performance status; n.a.: not available; loc. adv.: locally advanced; CR: complete response; PR: partial response; SD: stable disease; PD: progressive disease; PFS: progression-free survival; OS: overall survival; +: patient censored without event at the last observation.

**Table 3:** Main characteristics and results obtained in the studies with paclitaxel in patients with advanced angiosarcoma.

Author, year [ref]	Type of study	Paclitaxel dose and schedule	Period of treatment	Number of patients	Response rate (%)	PFS (median)	OS (median)
Fata et al., 1999 [20]	Retrospective	250 mg/m^2^ continuous infusion for 24 h every 3 weeks or 175 mg/m^2^ every 3 weeks or 90 mg/m^2^ weekly	1992–1998	9	89	TTP 5 months	n.a.

Schlemmer et al., 2008 [21]	Retrospective	135–175 mg/m^2^ every 3 weeks (*n* = 21) or 75–100 mg/m^2^ weekly (*n* = 11)	1996–2005	32	62	TTP 7.6 months	n.a.

Penel et al., 2008 [22]	Prospective	80 mg/m^2^ on days 1, 8, and 15, every 4 weeks	2005–2006	30 (assessable 27)	18-19	TTP 4 months	8 months

Penel et al., 2012 [9]	Retrospective	Weekly schedule	1996–2009	47	45	TTP 5.6 months	13.1 months

Italiano et al., 2012 [23]	Retrospective	80 mg/m^2^ on days 1, 8, and 15, every 4 weeks	1990–2010	75	53	5.8 months	10.3 months

Ray-Coquard et al., 2015 [24]	Prospective	Control arm: paclitaxel 90 mg/m^2^ on days 1, 8, and 15, every 4 weeks, for 6 cycles	2010–2013	26		PFS: 6.8 mo Progression-free rate at 6 months: 57%	Overall survival at 1 year: 55%
		Experimental arm: same as control arm + bevacizumab 10 mg/kg on days 1, 8, and 15 followed by maintenance therapy 15 mg/kg/3 wks until intolerance/progression	2010–2013	26		PFS: 6.9 mo Progression-free rate at 6 months: 57%	Overall survival at 1 year: 58%

Our study	Prospective + retrospective	80 mg/m^2^ on days 1, 8, and 15, every 4 weeks	Prospective: 2002–2006 Retrospective: 2003–2011	18 (17 evaluable)	35	4.6 months	18.6 months

PFS: progression-free survival; OS: overall survival; TTP: time-to-progression; n.a.: not available.
